# Bubble effect: including internet search engines in systematic reviews introduces selection bias and impedes scientific reproducibility

**DOI:** 10.1186/s12874-018-0599-2

**Published:** 2018-11-13

**Authors:** Marko Ćurković, Andro Košec

**Affiliations:** 1 University Psychiatric Hospital Vrapče, Bolnička cesta 32, Zagreb, Croatia; 2 Department of Otorhinolaryngology and Head and Neck Surgery, University Hospital Center Sestre milosrdnice, Vinogradska cesta 29, Zagreb, Croatia

**Keywords:** Literature searches, Reporting, Web searching, Research ethics, Scientific rigor

## Abstract

**Background:**

Using internet search engines (such as Google search) in systematic literature reviews is increasingly becoming a ubiquitous part of search methodology. In order to integrate the vast quantity of available knowledge, literature mostly focuses on systematic reviews, considered to be principal sources of scientific evidence at all practical levels. Any possible individual methodological flaws present in these systematic reviews have the potential to become systemic.

**Main text:**

This particular bias, that could be referred to as (re)search bubble effect, is introduced because of inherent, personalized nature of internet search engines that tailors results according to derived user preferences based on unreproducible criteria. In other words, internet search engines adjust their user’s beliefs and attitudes, leading to the creation of a personalized (re)search bubble, including entries that have not been subjected to rigorous peer review process. The internet search engine algorithms are in a state of constant flux, producing differing results at any given moment, even if the query remains identical. There are many more subtle ways of introducing unwanted variations and synonyms of search queries that are used autonomously, detached from user insight and intent. Even the most well-known and respected systematic literature reviews do not seem immune to the negative implications of the search bubble effect, affecting reproducibility.

**Conclusion:**

Although immensely useful and justified by the need for encompassing the entirety of knowledge, the practice of including internet search engines in systematic literature reviews is fundamentally irreconcilable with recent emphasis on scientific reproducibility and rigor, having a profound impact on the discussion of the limits of scientific epistemology. Scientific research that is not reproducible, may still be called science, but represents one that should be avoided. Our recommendation is to use internet search engines as an additional literature source, primarily in order to validate initial search strategies centered on bibliographic databases.

## Background

Transparency, reproducibility, and rigor were recently reemphasized as fundamental characteristics of scientific epistemology [1]. Historically, the scientific biomedical community canonized major ethical issues through the use of safeguards, such as informed consent, external independent review process, and the peer review process. However, issues regarding transparency and reproducibility could be seen as ethical issues per se, as they provoke questions regarding ends and means of science itself [2, 3]. In order to integrate the vast quantity of available knowledge, literature mostly focuses on systematic reviews, considered to be principal sources of scientific evidence at all practical levels [4]. Any possible individual methodological flaws present in these systematic reviews have the potential to become systemic [5].

## Main text

Many guidelines exist on how to methodologically plan, implement and report a search of biomedical scientific literature. Current standards of reporting on search methodology have been found lacking and certain standards have already been proposed [6, 7]. Almost none of them have addressed the bubble effect issue. That is a tendency to be selectively exposed to personalized information in a way that influences individual beliefs and attitudes [8]. Systematic literature reviews start with a bibliographic database search accessing a large number of peer-reviewed scientific studies. It has become increasingly frequent to include internet search engines as access points as well [6, 7, 9]. Internet search engines can be useful in reviewing literature not found in common bibliographic databases. The need for this kind of supplemental literature is mostly justified with producing more comprehensive and applicable outcomes of scientific research [7, 9]. Although internet search engines seem immensely useful, they produce multiple sources of bias, and are ultimately irreproducible, no matter how seriously one takes transparency and rigor into account. The end user, whoever that might be, is principally concerned with the usefulness of data, and often does not pay attention to the underlying search methodology [10]. Alternative trajectories of acquiring scientific knowledge are tempting, but add to the risk of bypassing one of the primary safeguards – including entries that have not been subjected to rigorous peer review process. Moreover, internet search engines tailor search results according to derived user preferences based on unreproducible criteria. Pariser introduced the term “filter bubble” in 2011, as a personal selection bias inherent to internet search engines [8]. Some internet search engines support the use of advanced search features such as Boolean logic, but are not adequate equivalents of bibliographic databases. In contrast, internet search engines use personalized algorithms in order to evaluate and stratify results according to the trustworthiness of websites, and relevance to the search query in comparison to the end user’s search history, among other things [8, 11]. The most common example of personalization is the redirection to a country specific search engine default version. There are many more subtle ways of adjusting for the user’s beliefs and attitudes, leading to the creation of a personalized search bubble. In addition, variations and synonyms of search queries are used autonomously, detached from user insight and intent [11]. The internet search engine algorithms are in a state of constant flux, producing differing results at any given moment, even if the query remains identical. Moreover, internet content is inherently unstable. Even the most well-known and respected systematic literature reviews do not seem immune to the some negative implications of the search bubble effect [6, 12–15]. Analyses have shown that internet searches implemented within Cochrane systematic reviews were not reported in sufficient detail to be transparent and reproducible [14]. Some of the most common issues found were inconclusive reporting on search queries and search limits, or a descriptive account of search methodology [14].

When using internet search engines for scientific purposes, some recommendations have been already stated, such as logging out of personal accounts, automatic or manual clearing of web search history, turning off web history options, using anonymous browsing options or using advanced search options [12]. Using speech marks or Verbatim options may reduce automatic reinterpreting of search queries as well as using meta-search engines that operate on different underlying settings (such as DuckDuckGo, Search Encrypt or StartPage). Simplification of retrieval and storage of scientific data from internet search engines has also been advised [16]. Even with regularly implementing these, systematic literature reviews using internet search engines remain vulnerable to the issue of reproducibility. Despite the fact that even systematic reviews that are based solely on bibliographic databases may not be entirely reproducible [17], using internet search engines has an profound, additional negative influence primarily on the processes of data searching, retrieval, storage and reporting of systematic literature reviews [6, 7, 9, 14, 15, 18]. Nonetheless, internet search engines are a beneficial method of reaching specific, predefined sources of data (such as sites of relevant agencies). Even used for those, obvious purposes, issues of data retrieval, storage and reporting of search methodology may persist.

The only solution at present, having in mind that internet search engines may be impossible to avoid as they provide valuable data that cannot be reached by other means, should be respect of the principle of transparency. Authors should disclose all relevant details of their search queries as well as their immediate context. Such an approach, in order to become relevant, demands a commitment from the entire scientific community. For now, using internet search engines and its associated lack of guidance in making internet searching reproducible fails to identify results without introducing bias. This practice may bring more harm than benefit.

Our recommendation is to use internet search engines as an additional literature source, primarily in order to validate and review initial search strategies centered on bibliographic databases. Internet search engines used with caution may be useful in the preparation phase of systematic reviews, to refine and create more robust bibliographic database search strategies. This may seem contradictory to general recommendations for conducting systematic review, which advise to search multiple databases and use additional search strategies [19, 20], but there is no such a thing as reproducible research when it is conducted by primarily unscientific means. In other words, scientific research that is not reproducible, may still be called science, but represents one that should be avoided. When facing the issue of reproducibility, one should at least make best effort to prevent, predict and finally to control possible harms. These commitments cannot be met when using internet search engines in systematic reviews as they are out of subjects reach.

## Conclusion

If we choose to encompass the entirety of knowledge, there is a good chance the (re)search bubble effect will lead us to results that have already been chosen for us. These (re)search bubble characteristics need to be addressed, as they are in stark contrast with transparency, reproducibility and rigor – the prerequisites of scientific thinking.
